# A self-centering and stiffness-controlled MEMS accelerometer

**DOI:** 10.1038/s41378-023-00647-4

**Published:** 2024-01-18

**Authors:** Yiming Jin, Zhipeng Ma, Ziyi Ye, Mingkang Li, Xudong Zheng, Zhonghe Jin

**Affiliations:** 1 School of Aeronautics and Astronautics, Zhejiang University, Hangzhou, 310013 China; 2 Key Laboratory of Micro/Nano-Satellite Research of Zhejiang Province, Hangzhou, 310007 PR China

**Keywords:** Electrical and electronic engineering, Sensors

## Abstract

This paper presents a high-performance MEMS accelerometer with a DC/AC electrostatic stiffness tuning capability based on double-sided parallel plates (DSPPs). DC and AC electrostatic tuning enable the adjustment of the effective stiffness and the calibration of the geometric offset of the proof mass, respectively. A dynamical model of the proposed accelerometer was developed considering both DC/AC electrostatic tuning and the temperature effect. Based on the dynamical model, a self-centering closed loop is proposed for pulling the reference position of the force-to-rebalance (FTR) to the geometric center of DSPP. The self-centering accelerometer operates at the optimal reference position by eliminating the temperature drift of the readout circuit and nulling the net electrostatic tuning forces. The stiffness closed-loop is also incorporated to prevent the pull-in instability of the tuned low-stiffness accelerometer under a dramatic temperature variation. Real-time adjustments of the reference position and the DC tuning voltage are utilized to compensate for the residue temperature drift of the proposed accelerometer. As a result, a novel controlling approach composed of a self-centering closed loop, stiffness-closed loop, and temperature drift compensation is achieved for the accelerometer, realizing a temperature drift coefficient (TDC) of approximately 7 μg/°C and an Allan bias instability of less than 1 μg.

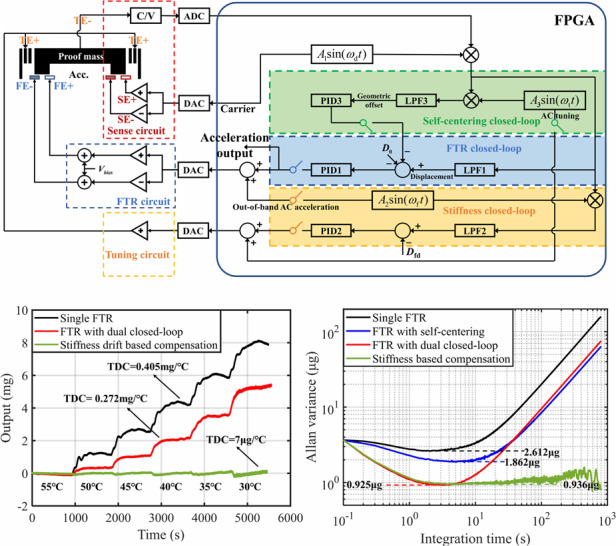

## Introduction

MEMS accelerometers have been widely used for consumer electronics, automotive, navigation, and vibration monitoring due to their small size, low cost, and low power consumption. For such high-performance applications as space and earth gravity measurements, high stability, and environmental adaptability are needed for accelerometers^1–5^. However, MEMS accelerometers suffer from severe temperature drift due to the temperature effect of their mechanical and electronic elements. Basic MEMS flexures composed of single-crystal silicon have a temperature-dependent Young’s modulus. Mismatched thermal expansion coefficients between layered MEMS structures and the package inevitably introduce thermal stress. The readout circuit of the MEMS accelerometers has a sensitivity that is dependent on the temperature variation. The suppression of temperature drift of the MEMS accelerometers becomes critical for long-term and high-performance measurement of acceleration.

Drift suppression has been realized by designing delicate stress-releasing, temperature-insensitive microstructures, and readout circuits and improving the manufacturing process. Studies on the temperature effect of the parasitic capacitance and the mechanical stiffness facilitate optimization of the mass-spring-anchor structure and the actuating/sensing capacitor^6,7^. He et al. proposed a design of middle-locating anchors to suppress the thermal stress of the MEMS accelerometer. Both the analytical model and measurements revealed an improvement in the temperature drift when inconsistent thermal expansion exists^8^. Similarly, Yin et al. reported a design of a temperature-insensitive structure to minimize the temperature drift of a MEMS accelerometer. This was achieved by designing stress-releasing anchor structures within the device and attaching the device to a die in a cantilever manner^9^. Ko et al. proposed a design of readout circuit topology with high programmability, showing a minimal temperature dependency^10^. The residue stress arising from mismatched thermal expansion, fabricated asymmetrical structures, and package integration has also been investigated to improve the fabrication and packaging process^11,12^. These methods provide valuable guidance for the improvement of the temperature drift in the design of accelerometers. However, their effectiveness in reducing temperature drift is limited due to not sufficiently compensating for existing residue stress.

Various temperature control and drift compensation approaches have been proposed for MEMS accelerometers. The most straightforward technique for eliminating the temperature drift is to maintain a constant temperature when operating the MEMS accelerometer. Micromachined ovens and microresonator thermometers have been proposed to realize a temperature-controlled miniatured MEMS system for accelerometers^13,14^. However, the integration of a temperature control system increases the complexity of the accelerometer system and sacrifices power consumption. Alternatively, the temperature-sensitive structures or the temperature-accompanying signals are utilized to compensate for the temperature drift of the MEMS accelerometer without increasing its power consumption and complexity. Compensation for temperature drift requires correlating the temperature drift with the temperature-dependent parameters calibrated beforehand, which requires additional calibration work. The accuracy of compensation depends on both the precision of calibration and the temperature model used. Microsized resonators such as the dual-ended tuning fork (DETF) are frequently used for calibrating the real-time temperature of MEMS gyroscopes and accelerometers. Their resonant frequency recognized by a phase-lock loop (PLL) is highly dependent on the operating temperature^15^. The capacitance of the as-fabricated capacitors as well as the parasitic resistance of accelerometers, are also used for calibrating the real-time temperature^16,17^. A novel approach for decoupling acceleration and temperature signals of resonant MEMS accelerometers has recently been proposed. This method is based on the different frequency shifts of two resonant modes arising from the effect of temperature and external acceleration^18^. Linear and polynomial models are commonly established to present the relationships of the accelerometer output drift and the temperature-calibrated parameters^19,20^, and they can be readily implemented for MEMS accelerometers. However, for the highly nonlinear temperature drift cases, the linear and polynomial models have limited accuracy. When dealing with highly nonlinear cases, artificial neural network approaches based on learning historical data have been proposed to improve the accuracy of drift compensation for accelerometers^21–23^, but they require a significant amount of training data and time.

MEMS accelerometers with high sensitivity and noise suppression were achieved by reducing the effective stiffness through geometric anti-spring flexures, electrostatic levitation, and electrostatic tuning^24–26^. The stiffness tuning of the MEMS accelerometer was devised based on the electrostatic spring softening of single/double-sided parallel plates (SSPP/DSPP)^27–29^. Although these stiffness-tuning accelerometers achieved low Allan bias instability under static conditions, their dynamic stability and suppression of temperature drift have not yet been fully addressed. The resulting bias arising from electrostatic tuning has not been fully eliminated for DSPP-based accelerometers, leading to severe temperature drift. This study improves the thermal adaptability and stability of MEMS accelerometers by employing DC/AC electrostatic tuning of DSPP. A self-centering/stiffness dual closed-loop approach was designed to enable automation in controlling the effective stiffness and determining the optimal reference position while compensating for temperature drift.

## Results and discussion

**Fig. 1:**
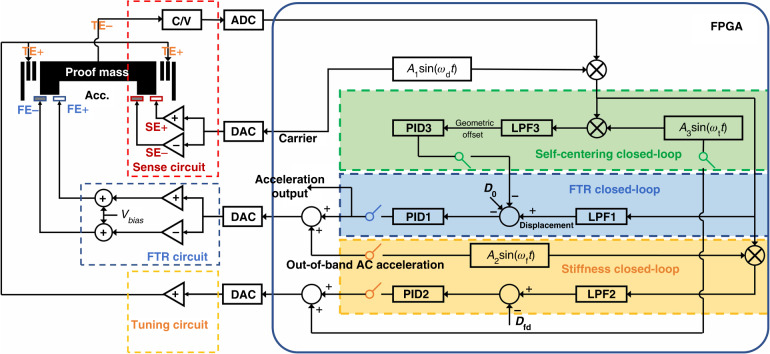
The schematic view of the proposed MEMS accelerometer system with self-centering/stiffness dual closed-loop. TE, FE, and SE are the tuning, the force feedback, and the sensing electrodes, respectively. PID1, PID2, and PID3 represent three controllers for FTR closed-loop, stiffness closed-loop, and self-centering closed loops, respectively; LPF1, LPF2, and LPF3 represent three low-pass filters of the demodulations in FTR closed-loop, stiffness closed-loop, and self-centering closed-loop, respectively; *D*_0_ and *D*_fd_ are the preset reference position of FTR closed loop and the preset reference stiffness of stiffness closed-loop, respectively

As illustrated in Fig. 1, the proposed novel MEMS accelerometer includes a two-layered proof mass and three sets of capacitors deployed for displacement sensing, force-to-rebalance (FTR), and electrostatic stiffness tuning. The accelerometer device was fabricated using a modified silicon-on-glass (SOG) process. The physical parameters of the fabricated accelerometer are summarized in Table 1. The controlling algorithm was implemented in a prototype platform comprising an analog circuit, the proposed accelerometer, and an FPGA-based digital circuit. The proposed controlling approach for the MEMS accelerometer was composed of three closed loops, including the FTR closed loop, the self-centering closed loop, and the stiffness closed loop. The FTR closed-loop preserved the proof mass at the reference position by applying a feedback electrostatic force, and the traditional PID and sliding-mode controllers of the FTR closed-loop were described in detail^28^. The self-centering closed-loop was implemented for the removal of the reference position error and the net electrostatic tuning forces caused by the drift of the readout circuit. The stiffness closed-loop described in previous work^29^ was introduced to keep constant the effective stiffness of the accelerometer.

**Table 1:** Physical properties of the accelerometer

Symbol	Quantity	Value
*ε*	Permittivity	8.85 × 10^−12^ F/m
*m*	Mass	8.49 mg
*N*	Number of the tuning capacitor pairs	62
*d*	The gap between the tuning capacitors	4 μm
*L*_*t*_	Overlapping length of the tuning capacitors	290 μm
*H*_*t*_	Overlapping width of the tuning capacitors	60 μm
*k*_*m*_	Mechanical stiffness	12.59 N/m
*c*	Viscous damping coefficient (30 °C)	0.0014 N m/s

In response to sufficient external acceleration, the capacitance change arising from the displacement of the proof mass can be detected by the sense circuit. The control error between the reference position and the actual position is fed to a PID controller, which produces a feedback voltage to the FTR circuit. The generated force of the FTR capacitors is applied to pull the proof mass back to its preset reference position. The feedback voltage output by the FTR closed-loop is utilized to represent its external acceleration. Therefore, the amplification gain of the C/V readout circuit causes the drift of the reference position with respect to the geometric center of DSPP under a temperature variation. By applying AC tuning to the DSPP capacitors, the offset from the geometric center can be detected in real-time. The corresponding temperature effect of the FTR reference can be eliminated by a self-centering closed loop. Simultaneously, a previously presented stiffness closed-loop approach is implemented to ensure a constant effective stiffness of the accelerometer even under temperature changes^29^.

### DC/AC electrostatic stiffness tuning

**Fig. 2:**
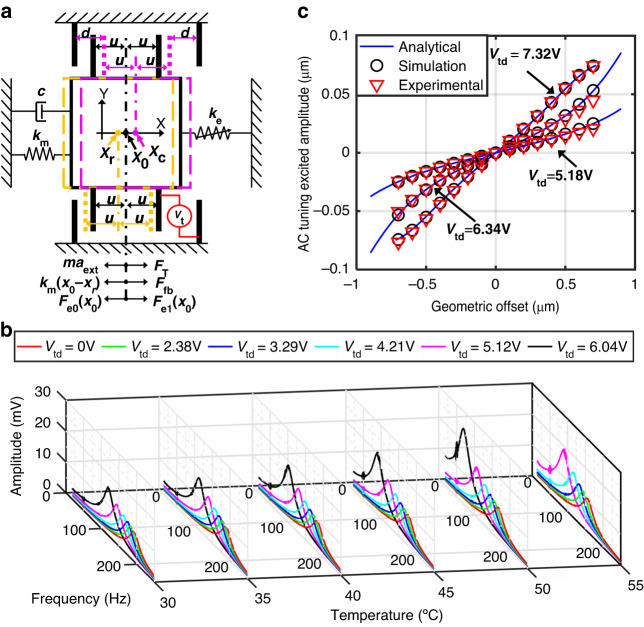
The results of the DC/AC electrostatic stiffness tuning. **a** Mechanical model of the accelerometer. *x*_r_, *x*_0_, *x*_c_ are the at-rest position, reference position of FTR, and geometric center, respectively; *d* is the designed gap of the tuning capacitors; *u* represents the distance from the movable tuning electrodes to the center of the proof mass. **b** The resonant amplitude-frequency curves of the accelerometer with different DC tuning voltages from 30 to 55 °C. **c** The measured and predicted in-phase amplitude of the AC tuning response under different geometric offsets and DC tuning voltages

A stiffness tuning principle based on applying a DC bias voltage to the capacitors of DSPP was detailed in a previous work^28^. An alternative stiffness tuning method based on applying an AC voltage to DSPP capacitors was implemented in this study. The dynamic model of DC/AC tuning is described in detail in Supplementary Note 1. As illustrated in Fig. 2a, the governing equation of motion of the stiffness-tuning accelerometer can be described as follows:

1 $$m\ddot{x}+c\dot{x}+{k}_{{\rm{m}}}(x-{x}_{{\rm{r}}})={F}_{{\rm{e0}}}+{F}_{{\rm{e1}}}+{F}_{{\rm{fb}}}+{F}_{{\rm{T}}}+m{a}_{{\rm{ext}}}$$

where *m*, *c*, and *k*_m_ are the mass, damping coefficient, and mechanical stiffness of the accelerometer, *F*_e0_ and *F*_e1_ are the DC and AC electrostatic tuning forces, *a*_ext_, *F*_T_, and *F*_fb_ are the input acceleration, residue stress, and FTR force, and *x* and *x*_r_ are the position and at-rest position of the proof mass, respectively. Notably, the at-rest position of the proof mass is inconsistent with the geometric center due to fabrication imperfection.

The proposed accelerometer was characterized using a frequency sweep experiment using FTR capacitors in a temperature-controlled chamber. The resonant amplitude–frequency curves of the proposed accelerometer are shown in Fig. 2b. As the temperature increases, the resonant frequency of the untuned accelerometer without electrostatic tuning is reduced. The reduction in the resonant frequency is attributed to the temperature effect of Young’s modulus of the single-crystal silicon flexures. The calibrated variation in the mechanical stiffness exhibited a quadratic relationship with the temperature. Alternatively, the resonant frequency of the proposed accelerometer can also be decreased by increasing the DC electrostatic tuning voltages. The resonant amplitude-frequency curve with the same tuning voltage could not be measured at 55 °C due to the occurrence of pull-in instability inferring a zero or negative effective stiffness.

The applied AC tuning voltage would have a much smaller amplitude than that of the DC tuning and a frequency out of the FTR bandwidth. The response of the AC stiffness tuning can be employed to calibrate the offset of the proof mass a technique from the geometric center between DSPPs. Hereafter, we defined the geometric center between the DSPP and the offset from the geometric center between the DSPP as the “geometric center” and the “geometric offset”, respectively. The total tuning voltage applied to DSPP capacitors is composed of an AC tuning voltage and a DC tuning voltage, expressed by

2 $${V}_{{\rm{t}}}={V}_{{\rm{td}}}+{V}_{{\rm{ta}}}\,\sin ({\omega }_{{\rm{t}}}t)$$

where *V*_t_, *V*_td_, and *V*_ta_ are the total tuning voltage, the DC tuning voltage, and the AC tuning voltage, respectively, and *ω*_t_ is the frequency of AC tuning. For simplicity, the PID controller of the FTR closed-loop is assumed to have robustness. Therefore, the stability of the proof mass under AC stiffness tuning is guaranteed. Because the applied AC tuning voltage is approximately 2 orders of magnitude less than the applied DC tuning voltage, the response of the AC tuning can be solved by the perturbation method, in which the steady-state response is first solved by neglecting the AC tuning and the perturbed response is solved by neglecting the second-harmonic AC electrostatic force and the AC stiffness terms. The in-phase AC response amplitude (*δ*) of the perturbation was derived as:

3 $$\delta =\frac{4{V}_{{\rm{td}}}{V}_{{\rm{ta}}}N\varepsilon {L}_{{\rm{t}}}{H}_{{\rm{t}}}d(x-{x}_{{\rm{c}}})}{m{[{d}^{2}-{(x-{x}_{{\rm{c}}})}^{2}]}^{2}\sqrt{{({{\omega }_{0}}^{2}-{{\omega }_{{\rm{t}}}}^{2})}^{2}+{(\frac{c}{m})}^{2}{{\omega }_{{\rm{t}}}}^{2}}}$$

where *ω*_0_ is the effective resonant frequency. The detailed solution process is presented in Supplementary Note 1, Eqs. (S9–S23). This equation indicates that the amplitude of the AC response is proportional to the geometric offset. The amplitude of the AC response can also be increased by increasing both the DC and AC stiffness tuning voltages. The AC electrostatic force should be much smaller than the DC electrostatic force.

The net electrostatic force resulting from DC/AC tuning can only be canceled out when the proof mass is rebalanced to the geometric center. Otherwise, the net electrostatic force is increased in proportion to the square of the tuning voltage. The geometric offset was assumed to be at least one order of magnitude smaller than the capacitive gap. The excited amplitude of AC tuning is predicted by the calculation using Eq. 3 or by a numerical simulation based on MATLAB/Simulink. The parameters of the solver used for simulation are listed in Table S1 of Supplementary Note 3. The accelerometer system parameters used for simulation are listed in Table 1, in which an AC tuning amplitude of 96 mV and a DC tuning voltage (7.32 V, 6.34 V, or 5.18 V) is applied to the accelerometer. The predictions were verified by measuring the accelerometer by deliberately varying the geometric offset. As shown in Fig. 2c, the predicted amplitudes as a result of AC tuning were in favorable agreement with the measured amplitudes. When the DC tuning voltage was 7.32V and the geometric offset was larger than ±0.72 μm, the effective stiffness of the accelerometer became zero, leading to pull-in instability. When the geometric offset was within the range of ±0.5 μm, the excited amplitude exhibited favorable linearity as a function of the geometric offset. To enhance the sensitivity of detecting geometric offset, both the AC and DC tuning voltages can be increased. However, to avoid pull-in instability and prevent excessive nonlinearity and deviation from the predicted model, both the AC and DC tuning voltages were only increased within a certain range. The sensitivity of detection is also influenced by the frequency of the excitation signal and the effective stiffness of the accelerometer.

### Self-centering closed loop

The effect of temperature on mechanical and circuit elements of another stiffness-tuning MEMS accelerometer with SSPP was previously characterized^29^. The drift from the mechanical stiffness and the gain of the readout circuit played a dominant role in the temperature drift of the accelerometer output. In this study, the temperature effect of the residue stress and the FTR reference were considered in the temperature drift model. To model the temperature of the residue stress, we measured the residue stress of the open-loop accelerometer at different temperatures without external acceleration. As shown in Fig. S1 of Supplementary Note 4, both the displacement and the residue stress of the open-loop accelerometer exhibit a quadratic tendency with temperature variation. Based on this characterization, the temperature effect of the residue stress is modeled by Eq. S5 in Supplementary Note 1. According to a previous characterization of the drift of the CV readout circuit at different temperatures^29^, the amplification gain exhibited a linear dependence on temperature. Therefore, the FTR reference is modeled by Eq. S25 in Supplementary Note 2. The temperature drift model of the proposed accelerometer is described in detail in Supplementary Note 2. By taking into account the temperature effect of residue stress, the mechanical stiffness, and the FTR reference position, the accelerometer output in digital form (*D*_fb_) when operated with the FTR closed loop was given by

4 $${D}_{{\rm{fb}}}(T)=-\frac{m{a}_{{\rm{ext}}}}{{k}_{{\rm{fb}}}}-\frac{{F}_{{\rm{T}}}(T)}{{k}_{{\rm{fb}}}}+\frac{{k}_{{\rm{m}}}(T)}{{k}_{{\rm{fb}}}}\left[\frac{{D}_{0}}{{k}_{{\rm{re}}}(T)}-{x}_{{\rm{r}}}\right]-\frac{N\varepsilon {L}_{{\rm{t}}}{H}_{{\rm{t}}}{{V}_{{\rm{t}}}}^{2}}{{k}_{{\rm{fb}}}}\frac{2d(\frac{{D}_{0}}{{k}_{{\rm{re}}}(T)}-{x}_{{\rm{c}}})}{{\left[{d}^{2}-{(\frac{{D}_{0}}{{k}_{{\rm{re}}}(T)}-{x}_{{\rm{c}}})}^{2}\right]}^{2}}$$

where *k*_re_ and *k*_fb_ are the gain of the readout and feedback circuit, respectively, and *D*_0_ is the reference position of the digital form in the FPGA. Equation 4 reveals that the output bias drift of the accelerometer is composed of the residue stress bias, the elastic force bias, and the electrostatic force bias. As illustrated in Eq. 4, the static output drift of the accelerometer is also dependent on the DC tuning voltage of DSPP due to the existing geometric offset. As the DC tuning voltage increases, the electrostatic tuning bias increases and even causes pull-in instability.

Based on the monotonic dependence of the excited amplitude on the geometric offset, a novel self-centering FTR controlling scheme was realized in the proposed accelerometer. A complimentary self-centering closed-loop with a conventional PID controller was incorporated into the conventional FTR closed-loop. This was developed to adaptively adjust the reference position of the FTR closed-loop until the excited amplitude reduces to zero. The self-centering closed-loop aims to position the proof mass at the right geometric center between DSPP locations, leading to the removal of the bias terms of the elastic force and the electrostatic tuning force as described in Eq. 4. and the output of the accelerometer when operated with self-centering closed-loop is obtained as:

5 $${D}_{{\rm{fb}}}=-\frac{m{a}_{{\rm{ext}}}}{{k}_{{\rm{fb}}}}-\frac{{F}_{{\rm{T}}}}{{k}_{{\rm{fb}}}}+\frac{{k}_{{\rm{m}}}}{{k}_{{\rm{fb}}}}({x}_{{\rm{c}}}-{x}_{{\rm{r}}})$$

**Fig. 3:**
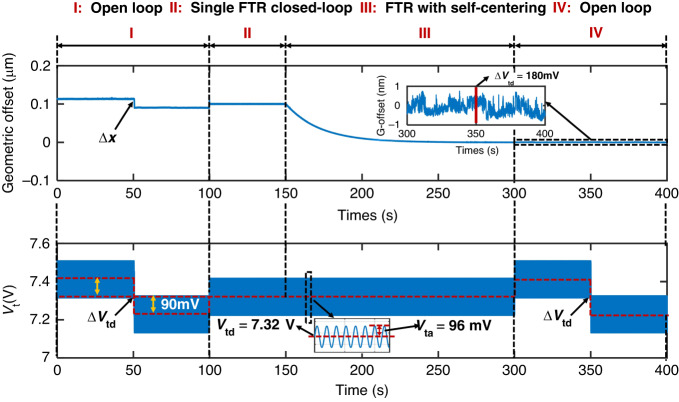
The measured geometric offset (upper figure) of the proposed accelerometer at different operation modes and DC/AC tuning states (lower figure). Without the self-centering closed-loop, the geometric offset is determined by the DC tuning voltage (Phase I, II, III). The self-centering closed loop enables the nulling of the geometric offset (Phase IV). In Phase V, the DC tuning voltage for the open-loop accelerometer is changed. The absence of change in the geometric offset verifies the accuracy of the self-adjusted geometric center

To demonstrate the effectiveness of the complementary self-centering closed loop, the accelerometer with both DC and AC tuning was tested with alternating operating modes, as shown in Fig. 3. AC electrostatic tuning with a voltage of 96 mV and a frequency of 113 Hz was applied to the proposed accelerometer. Due to the fabrication imperfections, the initial geometric offset of the DSPP capacitor exists, which was further increased upon applying a larger DC tuning voltage. Both DC and AC electrostatic tuning were first applied to the open-loop accelerometer. The initial geometric offset was approximately 0.1 μm, which can be altered by varying the DC tuning voltages for the accelerometer (Phase I). This is attributed to the residual forces of DC tuning, depending on the geometric offset and DC tuning voltage. The FTR closed-loop of the accelerometer was then rebalanced at a preset reference position (Phase II). In conventional cases, the preset reference position is designated with an unknown geometric offset. When applied to a self-centering FTR control scheme, the geometric offset is gradually reduced to zero (phase III). The accelerometer can be controlled to a self-adjusted center position automatically in Phase IV, and the accelerometer was operated in an open loop by applying a constant actuation force. The proof mass was, therefore, pulled to the predetermined center position. The position of the proof mass was unaltered even when varying the DC tuning voltage. This demonstrated that both the resulting DC and AC electrostatic forces were nulled when the proof mass was placed at the geometric center. Conventionally, the reference position of the FTR closed-loop was determined manually for the accelerometer and varied on a case-by-case basis. This indicates that the proposed self-centering approach enables not only the automatic adjustment of the FTR reference position but also the mitigation of the adverse effect of electrostatic tuning. The frequency (113 Hz) of AC tuning in the self-centering closed loop was selected by compromising the high signal-to-ratio and low interference of the acceleration sensing. To reduce the interference with the FTR closed-loop, an AC tuning frequency exceeding the FTR bandwidth should be applied, and an AC tuning frequency of approximately 113 Hz gives a relatively favorable signal-to-noise ratio.

**Fig. 4:**
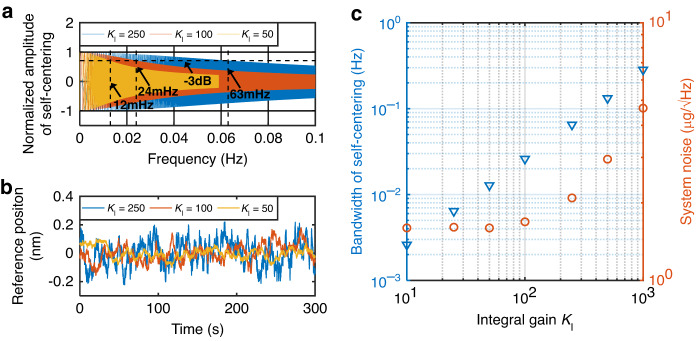
The bandwidth and resulted noise of the self-centering closed-loop. **a** The bandwidths of the self-centering closed loop with different integral gains of PID controller (*K*_I_). **b** Error of reference position introduced by the self-centering closed loop with different *K*_I_. **c** Summary for bandwidth of self-centering and the output noise of the accelerometer introduced by the self-centering closed loop with different *K*_I_

The self-centering closed-loop facilitates the automation of determining the FTR reference position, and additional errors were simultaneously introduced to the FTR closed-loop. To mitigate the related interference, the self-centering closed loop was designed with much less bandwidth than that of the FTR closed loop. This process was achieved by adjusting the integral gain of the PID controller. In this technique, the resulting error from the self-centering closed loop is minimized. The dynamic performance of the accelerometer operated with a self-centering FTR closed loop was evaluated by introducing an AC perturbation of the reference position. As shown in Fig. 4a, the bandwidths of the self-centering closed loop were modified by varying the integral gains *K*_I_ of the PID controller. The introduced error of the reference position for the static accelerometer under a self-centering FTR closed loop is shown in Fig. 4b. As the integral gains of the PID controller increased, both the bandwidth and the introduced reference error increased linearly, as shown in Fig. 4c. With the resulting increase in the integral gain of the PID controller in the self-centering closed loop, the self-centering bandwidth increases linearly, as shown in Fig. 4c. Although the noise of the acceleration output of the self-centered accelerometer is as low as 4 μg/√Hz when the integral gain is less than 100, the output noise is greatly increased when the integral gain exceeds 100. This indicates that there is a compromise between the bandwidth of the self-centering closed loop and the output noise of the self-centering accelerometer. To minimize the increase in noise due to the operation of the self-centering closed loop, we implemented that loop with a bandwidth of less than 24 mHz. DC electrostatic tuning with a voltage of 7.32 V and AC electrostatic tuning with a voltage of 96 mV with a frequency of 113 Hz were applied to the self-centering accelerometer.

By deploying the proof mass at the geometric center of DSPP, the self-centering closed-loop significantly reduces the electrostatic tuning force bias drift term described in Eq. 4 and can also suppress the output noise arising from the tuning voltage noise. However, due to the inevitable self-centering control error, as shown in Fig. 4b, the electrostatic tuning force bias in Eq. 4 cannot be reduced to zero. Therefore, the noise of the DC bias amplifies the self-centering error, degrading the performance of the accelerometer.

### Self-centering/stiffness dual closed-loop

**Fig. 5:**
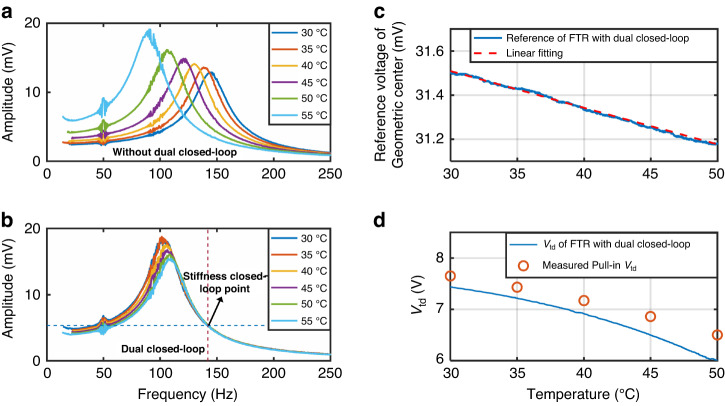
The temperature behaviors of the single FTR closed-loop and the stiffness/self-centering dual closed-loop. **a** The amplitude-frequency curves of a single FTR closed loop. **b** The amplitude-frequency curves of the stiffness/self-centering dual closed-loop. **c** Real-time reference voltage of geometric center when operating with dual closed-loop from 30 to 50 °C and its linear fitting. **d** Real-time DC tuning voltage and measured pull-in voltages when operating with dual closed-loop from 30 to 50 °C

The stiffness closed-loop approach has been previously proposed for an SSPP-based accelerometer to compensate for the temperature-dependent effective stiffness^29^. However, it has previously been proven that the temperature effect of the readout circuit, as well as the temperature effect of electrostatic tuning, cannot be fully eliminated with a stiffness-closed loop. In this study, the novel self-centering closed-loop and stiffness-closed loop were integrated, forming a self-centering/stiffness dual closed-loop scheme. With the self-centering closed loop, the temperature drift arising from the geometric offset can be mitigated. The introduction of the stiffness closed-loop enables stabilizing the accelerometer with low effective stiffness while preventing pull-in instability that often occurs due to temperature change, thereby improving the operating temperature range. The accelerometer in self-centering/stiffness dual closed-loop mode was measured by increasing the environmental temperature from 5 to 55 °C, as shown in Fig. S2 of Supplementary Note 5. This result indicates that the real-time electrostatic tuning voltage can be self-adjusted to have a lower value than that of the critical pull-in voltage, enabling an extension of the operating temperature range over 50 °C. Due to the orthogonal-axis stress effect, the mechanical stiffness has a 2nd-order polynomial dependency on the operating temperature. The effective stiffness can be consistently maintained by employing the dual closed-loop mode, regardless of both the temperature and stress effect of the mechanical spring. To demonstrate the performance of the proposed dual closed-loop scheme, the following temperature experiments were conducted within a range between 30 and 50 °C. As shown in Fig. 5a, the resonant frequency of the accelerometer with a single FTR closed loop was shifted from 145 to 90 Hz when the temperature was increased from 30 to 55 °C. The frequency shift of the resonant curve is attributed to the temperature drift of the mechanical component, including the temperature characteristics of Young’s modulus and the effect of internal stress. The noise at 50 Hz is attributed to the fundamental power frequency of the temperature-controlled chamber. When operated with a dual closed loop, the right-half side of the resonant response was matched even under a temperature change from 30 to 55 °C. The temperature-dependent shift of the resonant frequency of the accelerometer is greatly reduced by utilizing the stiffness closed loop. However, the frequency–response curves are tilted to the left in Fig. 5b, inferring that a weak nonlinearity arises from the stiffness closed loop. Because the accelerometer device was packaged with nitrogen, the quality factor of the squeeze film damping-dominated accelerometer is reduced when the temperature increases. The measured quality factors at different temperatures are presented in Table S2 in Supplementary Note 6. The reference point of the stiffness closed-loop was set on the right side of the frequency-response curve. This occurs because the response in the stiffness-closed loop varies monotonically in response to temperature. The variation in the quality factor with temperature causes a slight deviation of the left side of the resonant curve in Fig. 5b.

Both the FTR reference and the DC electrostatic tuning voltage can be automatically determined with the utilization of the self-centering and stiffness dual closed loop. To demonstrate the effectiveness of the dual closed-loop, the whole accelerometer system, including the sensor, FPGA chip, and circuits, was placed in the temperature-controlled chamber and subjected to a temperature variation from 30 to 50 °C. The critical pull-in voltages of the accelerometer at different temperatures were also measured at the predetermined geometric center position. As shown in Fig. 5c, the temperature-dependent geometric center was adjusted in real-time by the dual closed loop. The real-time FTR reference position can be well-fitted linearly, corresponding to the drift of the readout circuit. This result verifies the effectiveness of the linear model of the temperature effect of the FTR reference position. As the temperature of the uncentered accelerometer varies, both the mechanical stiffness and the electrostatic stiffness change with temperature, leading to a variation in the critical pull-in DC tuning voltage. The pull-in instability of the proposed accelerometer can only be prevented at a certain tuning range by optimizing the gains of the PID controllers. In this study, we focused on the self-centering and stiffness dual closed-loop approach for preventing pull-in instability. The critical pull-in voltages at which the effective stiffness becomes zero and, thus, pull-in occurs for the proposed accelerometer at different temperatures are listed in Table 2. Due to the inevitable vibration of the temperature chamber, the measured critical pull-in voltages were slightly lower than the predicted voltages. As shown in Fig. 5d, the DC electrostatic tuning was automatically adjusted to have a DC tuning voltage below the critical pull-in voltage by the dual closed-loop, showing the capability to prevent electrostatic pull-in instability and enhance the operating temperature range.

**Table 2:** Measured and predicted critical pull-in tuning voltages of the accelerometer at different temperature

Temperature (°C)	Measured pull-in voltage(V)	Standard deviation(mV)	Predicted pull-in voltage (V)
30	7.65	7.2	7.77
35	7.43	4.5	7.60
40	7.17	6.1	7.36
45	6.96	5.3	7.09
50	6.50	8.9	6.75
55	6.14	7.9	6.30

### Temperature drift compensation

When operated with the dual closed-loop scheme, the accelerometer still suffers from a temperature drift arising from the residue stress and elastic force, as described by Eq. 5. The remaining temperature drift *a*_d_ of the FTR with dual closed loops is expressed as:

6 $${a}_{{\rm{d}}}=-\frac{{F}_{{\rm{T}}}}{{k}_{{\rm{fb}}}}+\frac{{k}_{{\rm{m}}}}{{k}_{{\rm{f}}b}}({x}_{{\rm{c}}}-{x}_{{\rm{r}}})$$

**Fig. 6:**
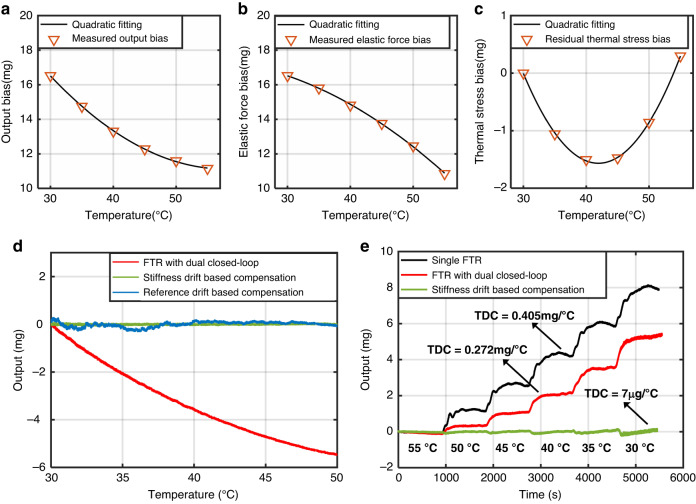
The temperature effect of the accelerometer under different control approaches. **a** Measured output bias from 30 to 55 °C and its quadratic fitting. **b** Measured elastic force bias and its quadratic fitting. **c** Measured residual thermal stress bias and its quadratic fitting. **d** Measured output of the accelerometer when operating with dual closed-loop and its compensation results based on two methods. **e** Temperature drift measurements and TDC results when operated with single FTR closed-loop, dual closed-loop, and stiffness drift-based compensation

To characterize the contribution of these loops, the accelerometer with a dual closed loop was warmed from 30 to 55 °C with an increment of 5 °C each time. Figure 6a shows a quadratic tendency of the output drift of the accelerometer with dual closed loops in response to temperature variation. The elastic force bias can, therefore, be obtained based on the measured mechanical stiffness at different temperatures, as shown in Fig. 6b. Therefore, the residue stress bias can be derived according to Eq. 6. As shown in Fig. 6c, the residue stress bias can be well described by a quadratic function of temperature. This verifies the effectiveness of the quadratic model of the temperature effect of the residue stress.

The results in Figs. 5 and 6 verify that the FTR reference position and the residue stress can be modeled by a linear and quadratic relationship with respect to temperature, respectively. Such a model is further utilized for temperature compensation of the self-centered and stiffness-controlled accelerometer. The linear dependence of the real-time calibrated geometric offset of the digital form on the temperature can be used to compensate for the residual bias drift. The remaining temperature drift *a*_d_ can be compensated by a quadratic function of the self-adjusted reference position. Alternatively, the stiffness closed-loop provides a real-time temperature-dependent quantity for compensating for residue bias drift. The remaining bias drift can also be compensated by a function of the DC stiffness tuning voltage, as obtained using:

7 $${a}_{{\rm{d}}}={f}_{2}{{V}_{{\rm{td}}}}^{2}+{f}_{1}\sqrt{{f}_{{\rm{sq}}}-{{V}_{{\rm{td}}}}^{2}}+{f}_{0}$$

where *f*_0_, *f*_1_, *f*_2_, and *f*_sq_ are the coefficients. Therefore, two temperature drift compensation approaches based on a self-centering reference and self-tuning DC voltage can be realized. Based on them, the accelerometer output can be corrected in operation by either the real-time self-adjusted reference or the DC tuning voltage. Hereafter, we defined these two temperature drift compensation methods as reference drift-based compensation and stiffness drift-based compensation, respectively.

As shown in Fig. 6d, both the reference drift- and stiffness drift-based compensation approaches exhibited a remarkable reduction in the temperature drift. The stiffness drift-based compensation approach was shown to have a better suppression of the temperature drift than the reference drift-based compensation approach for the accelerometer with dual closed loops. This can be attributed to a better signal-to-noise ratio of the controlled DC tuning voltage than the self-centering reference in terms of reflecting the temperature changes.

The temperature drift was measured for different controlling approaches under a temperature-controlled process, in which the temperature was first heated to 55 °C and then cooled to 30 °C with an interval of 5 °C every 15 min. Figure 6e shows a TDC of approximately 0.405 mg/°C when operated with a single FTR closed loop, a TDC of 0.272 mg/°C when operated with a dual closed loop, and a TDC of approximately 7 μg/°C when operated with a dual closed-loop and stiffness drift-based compensation approach. The self-centering/stiffness dual closed-loop approach was shown to facilitate the automation of mitigating the temperature drift arising from both the readout circuit and the mechanical element. Notably, the temperature effect of the residue stress of the proposed accelerometer might also be improved to some extent by minimizing residual stress due to temperature coefficient mismatch, such as using the SOI process^30,31^. With a self-centering closed loop, the temperature drift can be markedly reduced, as illustrated by Eq. 6. The temperature drift coefficient of 7 μg/°C corresponds to the temperature drift compensation error of the residue stress effect, which may require more precise higher-order compensation models.

Table 3 lists an overview of various temperature drift compensation methods for MEMS accelerometers. Compared to other methods, our method implemented an on-operation temperature compensation approach based on the developed temperature drift model or manual calibration, improving the thermal stability of the stiffness-tuning accelerometer.

**Table 3:** Comparison of temperature drift compensation methods

Reference	Method	Temperature drift coefficient	Limitation
^8^	Middle-located anchor	From 1.85 to 0.52 mg/°C	Limited improvement
^9^	Stress isolation design	Reduce to 1.9 μg/°C	Design complexity
^10^	Temperature-insensitive circuits	Reduce to 0.43 mg/°C	Increased circuit complexity
^12^	Low-stress packaging	Reduce to 10 mg/°C	Increased package cost
^13^	Micro oven-control system	From 185.9 mg/°C to 1.92 mg/°C	Increased power consumption
^15^	Integrated DETF resonator	From 1164 to 1.4 μg/°C	Additional structure
^16^	Parasitic resistance compensation	From 1.319 to 0.601 mg/°C	Limited improvement
^17^	Phase compensation	From 4.68 to 0.1 mg/°C	Deterioration of stability
^18^	Multiple Parameter Decoupling	N/A	Limitations on calibration accuracy
^20^	Resonant frequency compensation	From 3.54 to 0.05 mg/°C	Cross-coupling
^23^	Improved deep GRU	N/A	Requirements for training data and time
This work	Self-centering closed-loop and stiffness-based compensation	From 0.405 mg/°C to 7 μg/°C	Requirement for modeling or manual calibration

### Thermal stability performance

**Fig. 7:**
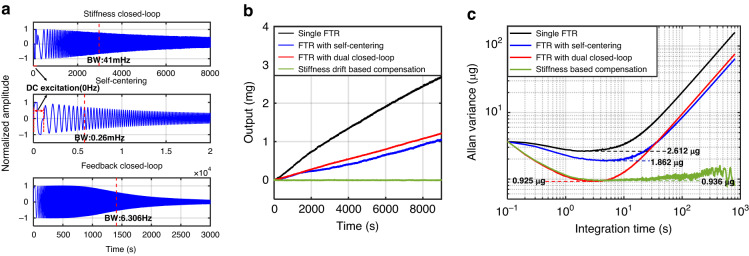
The performance of the accelerometer under different control approaches. **a** Bandwidth measurement of stiffness closed-loop, self-centering closed-loop and FTR closed-loop. **b** Measured output bias of the accelerometer during the natural cooling process. **c** Allan variances of the proposed accelerometer with different controlling approaches during the natural cooling process

With the proposed dual closed-loop and stiffness drift-based compensation approach, the stiffness-controlled and self-centering accelerometer is expected to have favorable thermal stability. The interference of the introduced dual closed loop is minimized by optimizing the out-of-band excitations and parameters of the low-pass filters and PID controllers. The FTR closed-loop was designed with a larger bandwidth than the dual closed loop to have better dynamical performance. This is reasonable because the working temperature in practice varies over the long term. Frequency-sweep experiments were carried out for each closed loop to evaluate their bandwidths. The amplitude of the frequency-varied sinusoidal response was normalized to that of the step response, as shown in Fig. 7a. The measured frequency at which the sinusoidal amplitude is reduced by 3 dB is defined as the bandwidth. The measured bandwidths of the stiffness closed-loop, the self-centering closed-loop, and the FTR closed-up for demonstration were 41 mHz, 0.26 mHz, and 6.206 Hz, respectively. Their bandwidths can be flexibly improved by increasing the integral gain of the corresponding PID controller. To verify the thermal stability, the accelerometer operated with different closed-loop modes was measured under a natural cooling process. This accelerometer was first heated to 40 °C and then kept for 30 min in a temperature-controlled chamber. The static measurement of the accelerometer was performed for 150 min after shutting down the heater/cooler of the chamber to prevent any influence from the vibration of the chamber. The reduction in the temperature during the natural cooling process was consistently approximately 8 °C, which is recorded in Supplementary Note 7. As shown in Fig. 7b, the accelerometer with a single FTR closed loop exhibits the largest temperature drift, which can be reduced to approximately half by the self-centering or dual closed-loop approach. The accelerometer that is operated with the dual closed-loop and stiffness drift-based compensation approach is shown to have two orders of magnitude of improvement in terms of suppressing the temperature drift.

The stability performance of the accelerometer was evaluated by an Allan variance analysis. The static output of the accelerometer was recorded with a sampling rate of 10 Hz during the same natural cooling process. The Allan variance data for different closed-loop modes are shown in Fig. 7c. The Allan bias instabilities of the accelerometer are approximately 2.612, 1.854, and 0.925 μg for single FTR closed-loop, self-centering closed-loop and dual closed-loop, respectively. This shows that the accelerometer with the self-centering closed loop has lower stability than that of the single FTR closed loop due to its capability of nulling the effect of electrostatic tuning and readout circuit. As reported before^27^, the Allan bias instability of the stiffness-tuning accelerometer is greatly improved by reducing the effective stiffness of the accelerometer. When subjected to a cooling process, the effective stiffness of the accelerometer would be increased in the case of a self-centering closed loop but maintained in the case of a dual closed loop. The control of constant low effective stiffness accounts for the improved Allan bias instability for the accelerometer with dual closed loops. The artificially introduced temperature variation during the testing process resulted in a significant temperature drift and degraded long-term performance for the uncompensated accelerometer. With the stiffness-based compensation method, the performance of the long-term variance can be remarkably improved. When the accelerometer was operated with a dual closed-loop and stiffness drift-based compensation approach, both the Allan bias instability and the long-term variance were greatly improved, as shown in Fig. 7c. This finding verifies the high stability performance of the proposed stiffness-controlled and self-centering accelerometer. The 1/*f* noise of the readout circuit was suppressed by modulating the displacement signal to a high frequency. The residual 1/*f* noise of the readout circuit is dominated by contributions from the carrier circuit, as shown in Fig. S4 in Supplementary Note 8. As the effective stiffness of the proposed accelerometer decreases, the equivalent noise within the FTR bandwidth can be further reduced, as illustrated in Fig. S5 in Supplementary Note 8.

The dynamic performance of the proposed accelerometer is limited by the stiffness-tuning DSPP. When the input acceleration exceeds the bandwidth or the maximum range, pull-in instability occurs due to the limited performance of the FTR closed loop. When the input acceleration is within the bandwidth, the maximum range of the proposed accelerometer is approximately ±280.4 mg. The maximum range attenuates when the input acceleration frequency exceeds the bandwidth. To address the problem of the limited dynamic performance of the proposed accelerometer, the effective stiffness can be kept at a positive value, while the proof mass can be controlled at the geometric center. This method is implemented in this study, improving the stability of the accelerometer system to some extent. An alternative method is to reduce the DC tuning voltage adaptively when the position control error of the FTR closed loop exceeds a threshold.

## Materials and methods

### Structural design

**Fig. 8:**
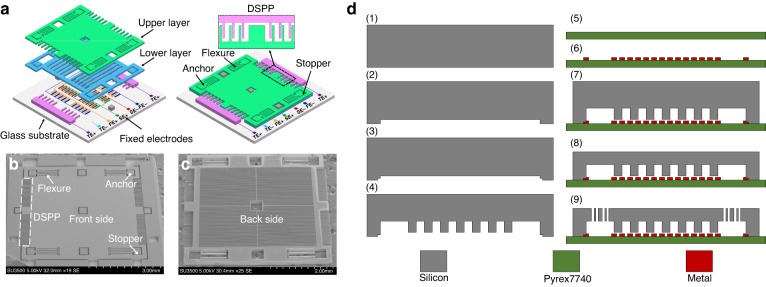
The design and fabrication of the proposed MEMS acceleroemter. **a** Schematic diagram of the accelerometer with two-layered proof mass. The upper layer has flexures, stiffness tuning electrodes, and stopper structure; the lower layer has displacement sensing and FTR electrodes. the proof mass is attached to the glass substrate through flexures and anchors. **b** Front-side SEM image of the fabricated accelerometer. **c** SEM image of the back-side of the fabricated proof mass. **d** Fabricating process of the accelerometer. (1) silicon cleaning, (2) 1st silicon patterning, (3) 2nd silicon patterning, (4) 3rd silicon patterning, (5) glass cleaning, (6) metal patterning, (7) anodic bonding, (8) silicon thinning, (9) structure releasing

As shown in Fig. 8a, the novel accelerometer is designed with four layers, including a layer of glass, a layer of metal, and two layers of silicon. The movable electrodes of the capacitors are attached to the proof mass and displaced against those counterpart fixed electrodes when subjected to external acceleration. The movable electrodes of the displacement sensing and FTR capacitors are designed on the lower layer of the proof mass. The overlapping capacitor areas between the movable and fixed electrodes vary linearly with respect to the displacement, and the capacitive gaps between the electrodes are held constant. The movable electrodes of the electrostatic stiffness tuning capacitors are designed on the upper layer of the proof mass. Their capacitive gaps vary together with the proof mass, producing a nonlinear electrostatic stiffness. To avoid pull-in failure between the parallel plates of the stiffness tuning capacitors, a stopper structure is designed between the proof mass and the anchors.

### Fabrication

The fabricated accelerometer devices are shown in Fig. 8b, c, and the fabrication process is illustrated in Fig. 8d. To improve the mechanical sensitivity, the proof mass is composed of two etched layers of silicon with the same thickness of 60 μm. The capacitive gap of 3 μm between the sensing and FTR capacitors was defined by UV lithography and deep reactive ion etching (DRIE) processes in Steps 1–2. A silicon pattern for the electrical connection between silicon and metal electrodes and a silicon electrode pattern is subsequently fabricated by repeating the UV lithography and DRIE processes in Steps 3–4. The etched depths of silicon are 200 nm and 60 μm. Steps 5–6 describe that the metal layers of Ti and Au with thicknesses of 30 nm and 200 nm, respectively, were subsequently sputtered on the glass wafer. The metal electrodes were patterned through UV lithography and reactive ion etching (RIE) processes. The silicon and glass wafers were bonded together by an anodic bonding process in Step 7. The silicon wafer was then thinned to 120 μm by a wet etching process in Step 8. Finally, as demonstrated in Step 9, the proof mass with flexures and the electrostatic tuning capacitors were fabricated by the UV lithography and DRIE processes. The etched thickness of the flexures and the tuning capacitors is approximately 60 μm.

### Algorithm implementation

Conventionally, the proof mass is rebalanced to the preset reference position when the accelerometer is operated with a single FTR closed loop. The proposed self-centering closed loop is realized to null the response of AC stiffness tuning based on adaptively adjusting the FTR reference position, as revealed in Eq. 3. The stiffness closed-loop is incorporated while keeping the response of an out-of-band acceleration excitation constant, which has been explained^29^. A schematic view of the controlling algorithm of the self-centering/stiffness dual closed loop is shown in Fig. 1. The controlling algorithm was implemented in a prototype platform composed of an analog circuit, the proposed accelerometer, and an FPGA-based digital circuit. Two out-of-phase carrier signals were applied to the fixed-sense electrodes. The displacement of the proof mass was picked up by a CV readout circuit. The error between the actual position and the preset reference position was sent to a PID controller. The feedback voltage representing the external acceleration was then generated for the feedback pull-push circuit. The DC/AC stiffness tuning voltage was produced by FPGA and then amplified by a circuit. The response of the AC stiffness tuning was acquired by the displacement sensing capacitor and the CV readout circuit. The amplitude of the AC tuning response was obtained by the first two demodulations with the carrier signal and the AC stiffness tuning signal. The in-phase component of the AC stiffness tuning response was sent to a PID controller to calculate the adjustment of the FTR reference position. In this technique, the proof mass can be controlled at the right geometric centering position between DSPP capacitors.

The self-centering closed-loop algorithm differs from the stiffness closed-loop algorithm in three aspects. The first difference is that AC excitation is applied to the FTR capacitor for the stiffness closed loop but to the DSPP capacitor for the self-centering closed loop. The second difference is that the response of the AC excitation is controlled to be a preset nonzero value for the stiffness closed-loop, yet to be zero for the self-centering closed-loop. The last difference is that the feedback is to adjust the DC stiffness tuning voltage for the stiffness closed-loop but to adjust the FTR reference position for the self-centering closed-loop. To avoid interference between the self-centering/stiffness closed loops, the frequencies of the two AC excitations should differ from each other and be much larger than the bandwidth of the FTR closed loop.

## Conclusion

A MEMS accelerometer with a self-centered reference position and an effective stiffness that is controlled to be constant is designed to achieve cutting performance of thermal stability. A dynamical model considering both electrostatic stiffness tuning and temperature drift was established, revealing the dominant effect of geometric offset. The temperature effect of the FTR reference and the residue stress were considered in the temperature drift models of both the uncentered and centered accelerometers. The temperature-dependent dynamic model with AC tuning and temperature drift was verified by measurements. A self-centering closed-loop approach was implemented to mitigate the effect of stiffness tuning based on AC electrostatic tuning of the DSPP capacitor. The stiffness-closed loop was also utilized to maintain the effective stiffness of the accelerometer, preventing electrostatic pull-in instability. With the self-centering closed loop, the temperature drift of the electrostatic tuning bias is nulled, preventing pull-in instability to some extent. The self-centering and stiffness dual closed loops were integrated into the conventional FTR closed loop, while their interference can be minimized by optimizing their bandwidths. The temperature-dependent elastic-force bias arising from the mismatch between the geometric center and the at-rest position of the proof mass and the temperature-dependent residue stress was revealed to account for the temperature drift of the accelerometer with dual closed loops. Two real-time temperature drift compensation methods were proposed based on the self-adjusted reference and electrostatic tuning voltage. The TDC of the accelerometer with the dual closed-loop and stiffness drift-based compensation scheme was reduced to 7 μg/°C, having a two-order of magnitude reduction compared to that of the single FTR closed loop. The bandwidths for stiffness closed-loop, self-centering closed-loop, and FTR closed-loop for demonstration were measured to be approximately 41 mHz, 0.26 mHz, and 6.206 Hz, respectively, which can be optimized in the future. The Allan variance analysis of the static output during a cooling process was performed for different closed-loop modes. The proposed accelerometer with dual closed-loop and stiffness drift-based compensation achieves the best stability performance, with an Allan bias instability of less than 0.936 μg and a long-term variance of approximately 1 μg. The results reveal that the proposed stiffness/self-centering dual closed-loop and compensation scheme can facilitate the control of the effective stiffness and enable suppression of the temperature drift and electrostatic pull-in instability. By deploying the proof mass at the geometric center of DSPP, the self-centering closed-loop significantly reduces the electrostatic tuning force bias drift term and can also suppress the output noise arising from the tuning voltage noise. However, due to the inevitable presence of self-centering control error, which is amplified by the self-centering error, the performance of the accelerometer was degraded. In addition, when the input acceleration exceeds the bandwidth or the maximum range, pull-in instability occurs due to the limited performance of the FTR closed loop. To address this problem, the effective stiffness can be conventionally kept at a positive value, while the proof mass can be controlled at the geometric center. This method improves the stability of the accelerometer system to some extent. An alternative method is to reduce the DC tuning voltage adaptively when the position control error of the FTR closed loop exceeds a threshold, which will be our future work.

### Supplementary information

Supplementary information
