# Supplementary material for: A self-centering and stiffness-controlled MEMS accelerometer
Source: Microsyst Nanoeng. 2024 Jan 18;10:11. doi: 10.1038/s41378-023-00647-4 (PMC10796430; doi:10.1038/s41378-023-00647-4)
Supplement: Supplementary file 1 — Supplementary information [file 41378_2023_647_MOESM1_ESM.docx]

# A self-centering and stiffness-controlled MEMS accelerometer: supplemental document

Yiming Jin^1,2^, Zhipeng Ma^1,2,^*, Ziyi Ye^1,2^, Mingkang Li^1,2^, Xudong Zheng^1,2^ and Zhonghe Jin^1,2^

^1^School of Aeronautics and Astronautics, Zhejiang University, Hangzhou, 310013, China

^2^Key laboratory of Micro/Nano-Satellite Research of Zhejiang Province, Hangzhou 310007, PR China

## Supplementary Note 1: Dynamic model of DC/AC tuning

### DC Electrostatic Stiffness Tuning

The stiffness tuning principle using the capacitors of DSPP was detailed in previous work^27^. The resulted electrostatic tuning force (*F*_e_) of DSPP capacitors when applied to a DC tuning voltage (*V*_td_) is given by

$F_{\text{e}}=N\varepsilon L_{\text{t}}H_{\text{t}}V_{\text{td}}^{2}\frac{2d(x-x_{\text{c}})}{[d^{2}-(x-x_{\text{c}})^{2}]^{2}}$ ,

where, *ε* is the permittivity, *N*, *L*_t_, *H*_t_ and *d* are number, overlapping length, overlapping width, and capacitive gap of DSPP, respectively, *x* and *x*_c_ are the position of the proof mass and geometric center position between DSPP, respectively. Hereafter, we defined the geometric center position between DSPP as “geometric center”.

And the electrostatic stiffness (*k*_e_) generated by DSPP is derived as

$k_{\text{e}}=-\frac{\partial F_{\text{e}}}{\partial x}=-N\varepsilon L_{\text{t}}H_{\text{t}}V_{\text{td}}^{2}\frac{2d^{3}+6d(x-x_{\text{c}})^{2}}{[d^{2}-(x-x_{\text{c}})^{2}]^{3}}$ .

As illustrated in Fig. 2a of the manuscript, the governing equation of motion of the stiffness-tuning accelerometer can be described as,

 ,

where, *m*, *c*, and *k*_m_ are mass, damping coefficient and mechanical stiffness of the accelerometer, *a*_ext_, *F*_T_ and *F*_fb_ are input acceleration, residue stress, and FTR force, *x*_r_ is at-rest position of the proof mass, which is inconsistent with the geometric center due to fabrication imperfection. The mechanical stiffness and residue stress were characterized to follow a quadratic polynomial function of temperature,

$k_{\text{m}}=a_{2}T^{2}+a_{1}T+a_{0}$ ,

$F_{\text{T}}=b_{2}T^{2}+b_{1}T+b_{0}$ ,

where, *a*_0_, *a*_1_, *a*_2_, *b*_0_, *b*_1_ and *b*_2_ are the coefficients of the quadratic polynomial functions and were calibrated in Fig. 2b and Fig. 6c of the manuscript.

When the accelerometer is operated with a FTR closed loop, the proof mass is controlled at the reference position. The first-order Taylor expansion of the electrostatic tuning force at the reference position is expressed as

$F_{\text{e}}(x)=F_{\text{e}}(x_{0})-k_{\text{e}}(x-x_{0})$ ,

where *F*_e_(*x*_0_) is bias force and *x*_0_ is reference position. By substituting Eq. (6) into (3), the governing equation can be rewritten as

 ,

where the effective stiffness is given by

$k_{\text{eff}}=k_{\text{m}}+k_{\text{e}}$ .

### AC Electrostatic stiffness tuning

An alternative stiffness tuning method by applying an AC voltage to DSPP capacitors is proposed in this study. The applied AC tuning voltage would have a much smaller amplitude than that of the DC tuning and a frequency out of the FTR bandwidth. The response of the AC stiffness tuning can be employed to calibrate the offset of the proof mass away from the geometric center between DSPP. Hereafter, we defined the offset from geometric center between DSPP as the “geometric offset”.

The total DC/AC stiffness tuning voltage applied to the DSPP capacitors is expressed as

$V_{\text{t}}=V_{\text{td}}+V_{\text{ta}}\sin(\omega_{\text{t}}t)$ ,

where *V*_t_ and *V*_ta_ are total tuning voltage and AC tuning voltage, respectively, *ω*_t_ is the frequency of the AC tuning. The resulted electrostatic force is composed of the DC term (*F*_e0_), first-harmonic AC term (*F*_e1_), and second-harmonic AC term (*F*_e2_), given by,

$F_{\text{e0}}=N\varepsilon L_{\text{t}}H_{\text{t}}\frac{2d(x-x_{\text{c}})}{[d^{2}-(x-x_{\text{c}})^{2}]^{2}}(V_{\text{td}}^{2}+\frac{1}{2}V_{\text{ta}}^{2})$ ,

$F_{\text{e1}}=N\varepsilon L_{\text{t}}H_{\text{t}}\frac{2d(x-x_{\text{c}})}{[d^{2}-(x-x_{\text{c}})^{2}]^{2}}2V_{\text{td}}V_{\text{ta}}\sin(\omega_{\text{t}}t)$ ,

$F_{\text{e2}}=N\varepsilon L_{\text{t}}H_{\text{t}}\frac{2d(x-x_{\text{c}})}{[d^{2}-(x-x_{\text{c}})^{2}]^{2}}\frac{1}{2}V_{\text{ta}}^{2}\cos(2\omega_{\text{t}}t)$ ,

respectively. The corresponding DC stiffness term (*k*_e0_), first-harmonic AC stiffness term (*k*_e1_), and second-harmonic AC stiffness term (*k*_e2_) are expressed as

$k_{\text{e0}}=-N\varepsilon L_{\text{t}}H_{\text{t}}\frac{2d^{3}+6d(x-x_{\text{c}})^{2}}{[d^{2}-(x-x_{\text{c}})^{2}]^{3}}(V_{\text{td}}^{2}+\frac{1}{2}V_{\text{ta}}^{2})$ ,

$k_{\text{e1}}=-N\varepsilon L_{\text{t}}H_{\text{t}}\frac{2d^{3}+6d(x-x_{\text{c}})^{2}}{[d^{2}-(x-x_{\text{c}})^{2}]^{3}}2V_{\text{td}}V_{\text{ta}}\sin(\omega_{\text{t}}t)$,

$k_{\text{e2}}=-N\varepsilon L_{\text{t}}H_{\text{t}}\frac{2d^{3}+6d(x-x_{\text{c}})^{2}}{[d^{2}-(x-x_{\text{c}})^{2}]^{3}}\frac{1}{2}V_{\text{ta}}^{2}\cos(2\omega_{\text{t}}t)$,

respectively. For simplicity, the PID controller of the FTR closed-loop is assumed with robustness. Therefore, the stability of the proof mass under a AC stiffness tuning is guaranteed. Since the applied AC tuning voltage is about 2-order of magnitude less than the applied DC tuning voltage, the response of the AC tuning can be solved by the perturbation method. Under a robust FTR and an AC stiffness tuning, the response of the proof mass is assumed as

$x=x_{0}+\delta$ ,

where *δ* is the position perturbance of the proof mass. The steady state solution in the absence of the AC electrostatic force is given by

$F_{\text{fb}}=-ma_{\text{ext}}-F_{\text{T}}+k_{\text{m}}(x_{0}-x_{\text{r}})-F_{\text{e0}}$ .

The governing equation of the perturbed proof mass by the AC electrostatic force can be expressed as

 .

For simplicity, the small quantities including the second-harmonic AC electrostatic force term, and the first-harmonic and second-harmonic AC electrostatic stiffness terms are omitted. The governing equation of motion of the perturbed proof mass by the first-harmonic AC electrostatic force is given by

 .

where the effective stiffness becomes

$k_{\text{eff}}=k_{\text{m}}+k_{\text{e0}}(x_{0})$ .

The effective resonant frequency is given by

$\omega_{0}=\sqrt{\frac{k_{\text{m}}}{m}-\frac{k_{\text{e0}}(x_{0})}{m}}$ .

The AC response of the perturbation can be solved as

 ,

where the normalized amplitude and the corresponding phase of transfer function *H*(*ω*_t_) are given by

$$\left|H(\omega_{\text{t}})\right|=\frac{1}{\sqrt{(\omega_{0}^{2}-\omega_{\text{t}}^{2})^{2}+(\frac{c}{m})^{2}\omega_{\text{t}}^{2}}}$$

and

 ,

respectively. It indicates that the amplitude of AC response is proportional to the geometric offset. The amplitude of AC response can also be increased by increasing both the DC and AC stiffness tuning voltages. It should be guaranteed that the AC electrostatic force is set to be much smaller than the DC electrostatic force.

and temperature drift model of the accelerometer

## Supplementary Note 2: Temperature drift model

The reference position of the FTR closed loop depends on the temperature drift of the readout circuit and can be expressed as

$D_{0}=k_{\text{re}}x_{0}$ ,

where *D*_0_ is the reference position of digital form in FPGA and *k*_re_ is the gain of the readout circuit. The gain drift of the readout circuit can be modeled by a linear function of temperature,

$k_{\text{re}}=c_{1}T+c_{0}$ ,

where c_0_ and *c*_1_ are the coefficients and were calibrated in Fig. 5c of the manuscript.

The temperature effect of mechanical and circuit elements of another stiffness-tuning MEMS accelerometer with SSPP was characterized^28^. It revealed that the drift from the mechanical stiffness and the gain of the readout circuit played a dominant role in the temperature drift of the accelerometer output. In this study, the temperature effect of the residue stress and the FTR reference were taken into account in the temperature drift model. By substituting Eq. (25) into Eq. (17), the accelerometer output in digital form can be given by

$\begin{matrix} D_{\text{fb}}(T)=-\frac{ma_{\text{ext}}}{k_{\text{fb}}}-\frac{F_{\text{T}}(T)}{k_{\text{fb}}}+\frac{k_{\text{m}}(T)}{k_{\text{fb}}}[\frac{D_{0}}{k_{\text{re}}(T)}-x_{\text{r}}]- \\ \text{ }\frac{N\varepsilon L_{\text{t}}H_{\text{t}}V_{\text{t}}^{2}}{k_{\text{fb}}}\frac{2d(\frac{D_{0}}{k_{\text{re}}(T)}-x_{\text{c}})}{[d^{2}-(\frac{D_{0}}{k_{\text{re}}(T)}-x_{\text{c}})^{2}]^{2}} \end{matrix}$,

where *k*_fb_ is the gain of the feedback circuit. Eq. (27) reveals that the output bias drift of the accelerometer is composed of the residue stress bias, the elastic force bias, and the electrostatic force bias.

As revealed in Eq. (22), the amplitude of the AC response is proportional to the first-order harmonic electrostatic force. It can only be nulled when the proof mass is placed at the geometric center position between the DSPP capacitors. The nulling of the AC electrostatic tuning is independent on the temperature. As a result, an adaptive FTR closed-loop can be realized by introducing a self-centering closed-loop. The reference position of FTR closed loop would be self-adjusted to approach the geometric center of DSPP, at which the response of the AC stiffness tuning reaches zero. Under the self-centering closed-loop, the target reference position of digital form can be given by

$D_{\text{c}}=k_{\text{re}}x_{\text{c}}$ .

By replacing *D*_0_ with *D*_c_ in Eq. (27), the accelerometer output becomes

$D_{\text{fb}}=-\frac{ma_{\text{ext}}}{k_{\text{fb}}}-\frac{F_{\text{T}}}{k_{\text{fb}}}+\frac{k_{\text{m}}}{k_{\text{fb}}}(x_{\text{c}}-x_{\text{r}})$ .

As indicated in Eq. (29), the temperature effect arising from the drift of the readout circuit can be eliminated by the proposed self-centering closed-loop. The residue stress bias and the elastic force bias remain for the proposed accelerometer. The remaining temperature drift *a*_d_ can be expressed as

$a_{\text{d}}=-\frac{F_{\text{T}}}{k_{\text{fb}}}+\frac{k_{\text{m}}}{k_{\text{f}b}}(x_{\text{c}}-x_{\text{r}})$ .

The real-time temperature dependent quantity provided by the digital form of geometric center can be used for compensating the residual bias drift. By combining Eq. (4,5), (26) and (28), the remaining temperature drift *a*_d_ can be represented by a quadratic function of the self-adjusted reference position, given by

 ,

where *e*_0_, *e*_1_ and *e*_2_ are the coefficients. The stiffness-closed loop was proposed for another stiffness-tuning MEMS accelerometer with SSPP to reduce the temperature effect of the mechanical stiffness^28^. The effective stiffness of the MEMS accelerometer is controlled to be constant by adjusting the DC electrostatic tuning voltage according to the response of an out-of-band excitation. By introducing the stiffness-closed loop in this study, the temperature drift of the mechanical stiffness is canceled out by adjusting the electrostatic stiffness. The effective stiffness becomes

$k_{\text{eff}}=k_{\text{m}}(T)-N\varepsilon L_{\text{t}}H_{\text{t}}V_{\text{td}}^{2}(T)\frac{2d^{3}+6d(\frac{D_{0}}{k_{\text{re}}(T)}-x_{\text{c}})^{2}}{[d^{2}-(\frac{D_{0}}{k_{\text{re}}(T)}-x_{\text{c}})^{2}]^{3}}$.

When operated with both the self-centering closed loop and the stiffness-closed loop, the effective stiffness becomes

$k_{\text{eff}}=k_{\text{m}}(T)-\frac{2N\varepsilon L_{\text{t}}H_{\text{t}}V_{\text{td}}^{2}(T)}{d^{3}}$ .

The DC stiffness tuning voltage is temperature-dependent and derived as

$V_{\text{td}}(T)=\sqrt{\frac{[k_{\text{m}}(T)-k_{\text{eff}}]d^{3}}{2N\varepsilon L_{\text{t}}H_{\text{t}}}}$ .

It is noted that the stiffness-closed loop cannot eliminate the elastic force bias. However, it provides a real-time temperature-dependent quantity for compensating the residue bias drift. By combining Eq. (4,5) and (33), the remaining bias drift can be modeled as a function of the DC stiffness tuning voltage, given by

$a_{\text{d}}=f_{2}V_{\text{td}}^{2}+f_{1}\sqrt{f_{\text{sq}}-V_{\text{td}}^{2}}+f_{0}$ ,

where *f*_0_, *f*_1_, *f*_2_ and *f*_sq_ are the coefficients. Based on Eq. (31) and (35), we proposed two different methods for compensating the bias drift of the accelerometer using the real-time self-adjusted reference position and the DC stiffness tuning voltage in this study.

## Supplementary Note 3: The Simulink solver parameters.

| **Table S1**  The Simulink solver parameters | |
| --- | --- |
| Solver | Ode45(Dormand-Prince) |
| Max step size | 1e-5 |
| Min step size | 1e-6 |
| Initial step size | 1e-6 |
| Relative tolerance | 1e-4 |
| Absolute tolerance | 1e-5 |
| Number of consecutive min steps | 1 |

## Supplementary Note 4: Open-loop position of the proof mass at different temperature without external acceleration.


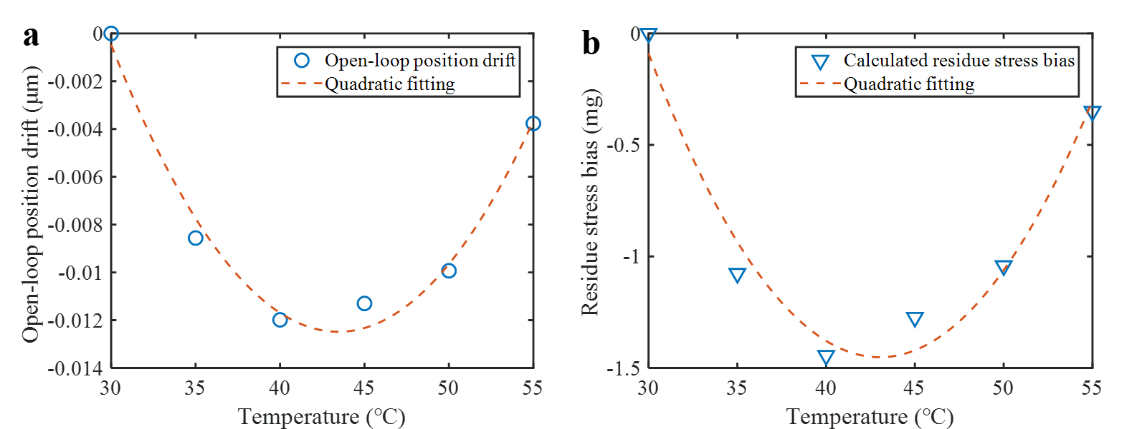


**Fig. S1**. **a** Open-loop position drift of the accelerometer at different temperature. **b** Calculated residue stress bias.

## Supplementary Note 5: The real-time electrostatic tuning voltage of the accelerometer with self-centering/stiffness closed-loop mode and the measured/predicted critical pull-in voltages.


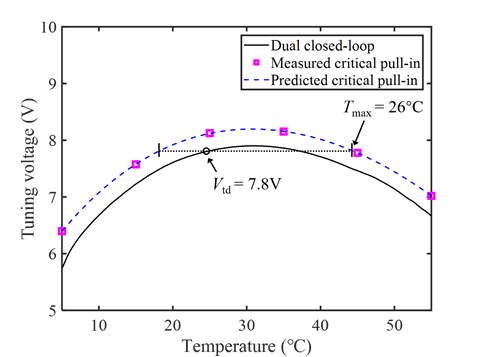


Fig. S2. The real-time electrostatic tuning voltage of the accelerometer with self-centering/stiffness closed-loop mode and the measured/predicted critical pull-in voltages. For comparison, the operating temperature range (*T*_max_) of the accelerometer with a constant electrostatic tuning voltage of 7.8 V and an FTR closed-loop is predicted to be about 26 °C.

## Supplementary Note 6: Temperature records of cooling process for single FTR, FTR with self-centering and FTR with dual closed-loop


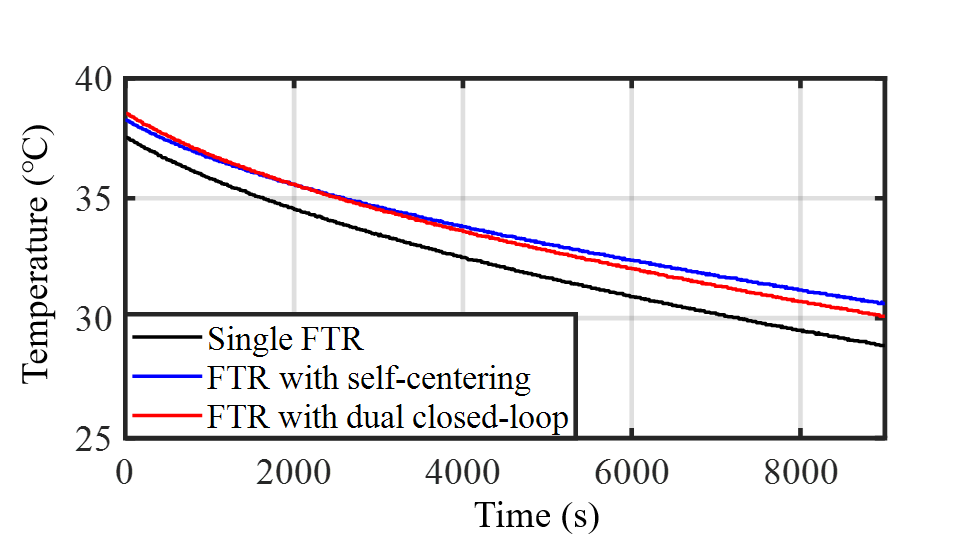


**Fig. S3** Temperature records of cooling process for single FTR, FTR with self-centering and FTR with dual closed-loop

## Supplementary Note 7: The measured quality factors at different temperatures.

**Table S2**

The measured quality factors at different temperatures

| Temperature(℃) | Quality factor |
| --- | --- |
| 30 | 7.38 |
| 35 | 7.20 |
| 40 | 6.87 |
| 45 | 6.53 |
| 50 | 6.14 |
| 55 | 5.64 |

## Supplementary Note 8: The measured quality factors at different temperatures.

|  |
| --- |
| **Fig. S4.** Measured noise spectra of the carrier circuit and the entire readout circuit. |

|  |
| --- |
| **Fig. S5.** The noise spectrum of the equivalent acceleration caused by the 1/f noise at different effective stiffness |
